# Advanced Glycation End Products: New Clinical and Molecular Perspectives

**DOI:** 10.3390/ijerph18147236

**Published:** 2021-07-06

**Authors:** Juan Salazar, Carla Navarro, Ángel Ortega, Manuel Nava, Daniela Morillo, Wheeler Torres, Marlon Hernández, Mayela Cabrera, Lissé Angarita, Rina Ortiz, Maricarmen Chacín, Luis D’Marco, Valmore Bermúdez

**Affiliations:** 1 Endocrine and Metabolic Diseases Research Center, School of Medicine, University of Zulia, Maracaibo 4004, Venezuela; jjsv18@gmail.com (J.S.); cpnm24@gmail.com (C.N.); angelort94@hotmail.com (Á.O.); manuelnava_14@hotmail.com (M.N.); wheelertorres16@gmail.com (W.T.); 2 Faculty of Medicine, School of Medicine, University of Buenos Aires, Buenos Aires 1121, Argentina; danielalemv@gmail.com; 3 Faculty of Medicine, School of Medicine, University of Zulia, Maracaibo 4004, Venezuela; marlonjh79@gmail.com; 4 City of Houston Health Department, Houston, TX 77054, USA; mayela.cabreradebravo@houstontx.gov; 5 Escuela de Nutrición y Dietética, Facultad de Medicina, Universidad Andrés Bello, Sede Concepción 4260000, Chile; lisse.angarita@unab.cl; 6 Facultad de Medicina, Universidad Católica de Cuenca, Cuenca 010105, Ecuador; rortiz@ucacue.edu.ec; 7 Facultad de Ciencias de la Salud, Universidad Simón Bolívar, Barranquilla 080002, Colombia; m.chacin@unisimonbolivar.edu.co; 8 Department of Nephrology, Hospital Clinico Universitario de Valencia, INCLIVA, University of Valencia, 46010 Valencia, Spain

**Keywords:** advanced glycation end products, diabetes mellitus, chronic complications, skin fluorescence

## Abstract

Diabetes mellitus (DM) is considered one of the most massive epidemics of the twenty-first century due to its high mortality rates caused mainly due to its complications; therefore, the early identification of such complications becomes a race against time to establish a prompt diagnosis. The research of complications of DM over the years has allowed the development of numerous alternatives for diagnosis. Among these emerge the quantification of advanced glycation end products (AGEs) given their increased levels due to chronic hyperglycemia, while also being related to the induction of different stress-associated cellular responses and proinflammatory mechanisms involved in the progression of chronic complications of DM. Additionally, the investigation for more valuable and safe techniques has led to developing a newer, noninvasive, and effective tool, termed skin fluorescence (SAF). Hence, this study aimed to establish an update about the molecular mechanisms induced by AGEs during the evolution of chronic complications of DM and describe the newer measurement techniques available, highlighting SAF as a possible tool to measure the risk of developing DM chronic complications.

## 1. Introduction

Diabetes Mellitus (DM) is a metabolic disease characterized by chronic hyperglycemia due to absent or inadequate insulin secretion, combining with defective action on target tissues, depending on the type of diabetes [1]. DM has many categories; however, the main subtypes are type 1 diabetes mellitus (T1DM), type 2 diabetes mellitus (T2DM), and gestational diabetes mellitus [2]. Diabetes is currently considered one of the largest epidemics of the twenty-first century. In 2015, according to the International Diabetes Federation (IDF), 415 million people worldwide were estimated to have diabetes, and there were approximately 5 million deaths attributable to diabetes, which is estimated as a death every 6 s [3]. However, it is to emphasize that the leading cause of death is not diabetes per se but DM-derived complications that lead to systemic dysfunction [4].

As a result, the prompt identification of DM-associated complications has become significantly relevant. DM complications can be divided into two main types, acute and chronic complications. Acute complications involve hypoglycemia and hyperglycemic crises, which tend to onset abruptly [5], instead of the slow and steady progression of chronic complications over the years [6]. Regardless of the type of diabetes mellitus, the chronic complications are likewise divided into two categories depending on the vascular damage. Macrovascular complications include cardiovascular disease (CVD); meanwhile, microvascular complications include chronic kidney disease (CKD), neuropathy, and diabetic retinopathy [7].

Diverse studies have determined that advanced glycation end products (AGEs) are involved in the pathophysiological mechanisms of DM complications [8]. These derive from nonenzymatic reactions between carbohydrate residues and protein, lipids, or nucleic acids, along with oxidative processes [9]. The development mechanism of DM complications through AGEs is varied, since these can generate structural changes to different macromolecules, altering their function and leading to intracellular pathways that trigger inflammatory responses and endothelial damage [10]. Thus, measuring these changes works both as a diagnostic method of DM and as a biochemical marker of glycemic control, being the determination of hemoglobin A1c (HbA1c), the most known marker tested [11].

The quantification of AGEs can also serve to assess the risk of developing DM complications and measure their degree of progression [9]. Although multiple measurement techniques have been developed, the search for more accurate, selective, and safe procedures is still ongoing [11]. Luckily, skin fluorescence (SAF) has recently been described in several studies as a noninvasive method [12]. Here, we analyze the pathophysiological mechanisms induced by AGEs that trigger the progression of chronic complications of DM and describe the newer measurement techniques available, focusing on SAF, a possible tool to measure the risk of developing DM complications.

## 2. Protein Glycation and Formation of Advanced Glycation End Products

Advanced glycation end products (AGEs) are an heterogeneous group of oxidative molecules with pathogenic capability [13]. The synthesis of these compounds begins with common metabolic pathways that occurred during the storage of food-derived products in the organism due to nonenzymatic reactions between reduced carbohydrates and free amino acids, peptides, lipids, or nucleic acids [14]. These reactions mentioned earlier are called Maillard reaction, glycation, or “nonenzymatic glycosylation” [15].

The generation of AGEs is completed through three stages [16], starting with the production of Schiff Bases [17], which emerge from the covalent bond established between the amino group of the free amino acid (generally composed by lysine and less frequently by arginine and cysteine residues), lipids, and nucleic acids with glucose [18,19]. This stage takes place in a time-lapse of hours following the postprandial glycemic increase, and it is characterized as a reversible reaction, since it can be re-established if the glycemic levels decrease [13].

**Figure 1:**
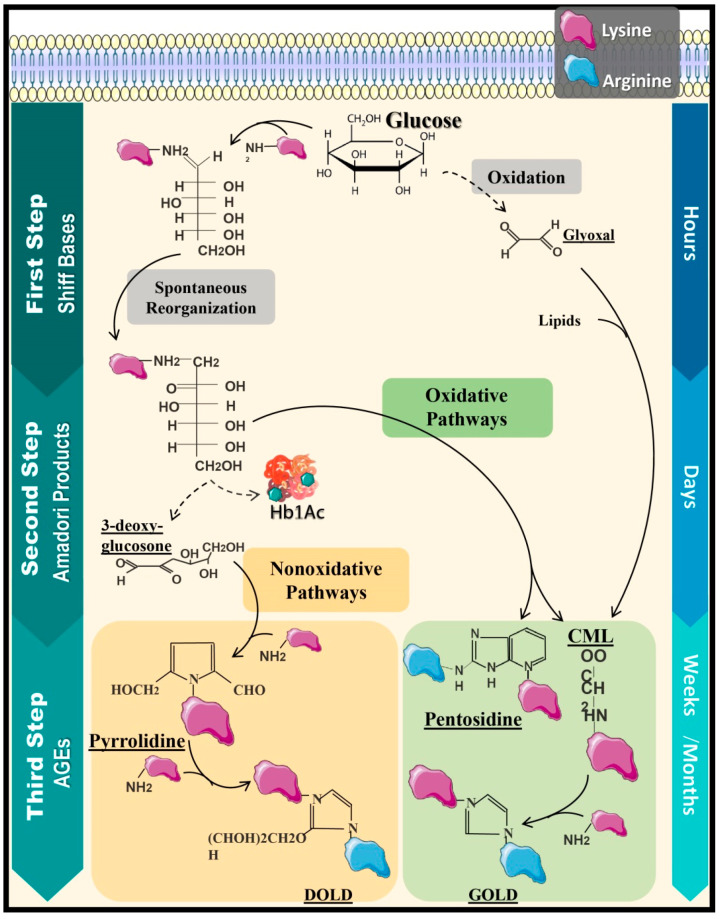
Maillard Reaction: production of AGEs. Production of AGEs initiates from a three-staged metabolic process denominated by a Maillard reaction, starting with the reversible formation of Schiff Bases, followed by a molecular rearrangement that results in the generation of Amadori products and, finally, leading to the formation of AGEs due to a posterior binding to secondary proteins, establishing as nonreversible structures. This can occur due to oxidative processes such as the formation of pyrrolidine and, also, nonoxidative processes such as the formation of pentosidine and CML; however, other proteins can be added to these molecules, allowing the formation of products such as DOLD and GOLD. Additionally, there are alternative pathways to form AGEs; for instance, the oxidation of glucose and subsequent glyoxal formation can generate CML by the binding of glyoxal to lipids. Hb1Ac: glycated hemoglobin and CML: 3,4-*N*-carboxymethyl-lysine.

Afterward, Schiff Bases submit to a molecular rearrangement, generating Amadori products, which are more stable compounds, although this stage is still reversible from carbohydrate oxidation [20]. The most recognized product is HbA1_c_, assembled through the junction of a valine residue of one of the β chain of this hemeprotein with plasma glucose [11]. Later, Amadori products accumulate in the organism; these go through reduction–oxidation reactions to eventually associate with secondary proteins through covalent bonds, altering their tertiary and quaternary structures and forming AGEs such as 3,4-*N*-carboxymethyl-lysine (CML), 3-deoxyglucosone (3DG), and Methylglyoxal (MG) [18]. AGEs have a brown-yellowish pigmentation, and some of them even have fluorescent properties, especially pyrrolidine, CML, imidazoline, and pentosidine [21]. Under physiological conditions, the glycation process occurs in weeks to years; nonetheless, in some pathological states such as hyperglycemia, oxidative stress, and temperature increase, the needed time can be reduced to hours [22] (Figure 1).

Comparatively, glucose has the slowest glycation rate of carbohydrate within these compounds, unlike fructose, glucose 6-phosphate, and threose (intracellular carbohydrates), which have a faster oxidation capacity [15]. Additionally, the formation of AGEs is also exogenous, since they can be found in food, like primarily animal-derived high-fat food such as beef [14]. Different studies have demonstrated that these types of food induce an increase in plasma levels of AGEs, since up to 10% of food rich in AGEs is absorbed into the bloodstream [13]. Despite the existence of discrepancies regarding the effects of exogenous compounds, studies have shown that a higher consumption of these food is correlated with weight gain [23], insulin sensitivity alteration [24], and albuminuria [25]. Therefore, AGE-rich diets, favoring oxidative stress and chronic inflammation states due to interactions with cellular compounds, are a substantial risk factor to the development of metabolic and cardiovascular complications [26].

## 3. AGEs and Their Implication in Chronic Complications of Diabetes Mellitus

Chronic complications of DM derive from structural and functional modifications of blood vessels due to hyperglycemia, affecting the heart, kidneys, and nervous system [27]. Among the mechanisms implied in developing these complications are the structural modifications induced by AGEs in vulnerable molecules such as proteins, lipids, and DNA, altering their stability and functions [28].

Activation of the receptor for advanced glycation end products (RAGE) represents the main mechanism involved between DM and AGEs [8]. RAGE is a cell surface receptor composed of three extracellular domains, a transmembrane domain, and a cytoplasmic tail [8], which belongs to the immunoglobulin superfamily [29], since its expression derives from the major histocompatibility complex class III (MHC-III) [30]. Thus, RAGE expression predominates in specific cells such as monocytes, macrophages, proximal tubular cells, podocytes, and mesangial cells [29].

As a result of the AGE–RAGE interaction, the cytoplasmic domain of RAGE leads to different signaling pathways. In particular, it can activate the p21 protein [31], triggering other signaling compounds to eventually stimulate kinases such as the extracellular signal-regulated kinase (ERK), c-Jun *N*-terminal kinase (JNK), and mitogen-activated protein kinase (MAP) [29], along with Janus kinase 1 and 2/Signal transducer and activators of transcription (JAK/STAT1) [32]. Finally, the consequences of these signal transduction pathways consist of the activation of transcription factors such as nuclear factor kappa B (NF-kB) [33] and the interferon-sensitive response element (ISRE) [34], which lead to the synthesis of proinflammatory cytokines as tumor necrosis factor-alpha (TNF-α) [34] and interleukins (IL) 1, 6, and 17 [35,36], as well as vascular cell adhesion molecule-1 (VCAM-1) [29].

**Figure 2:**
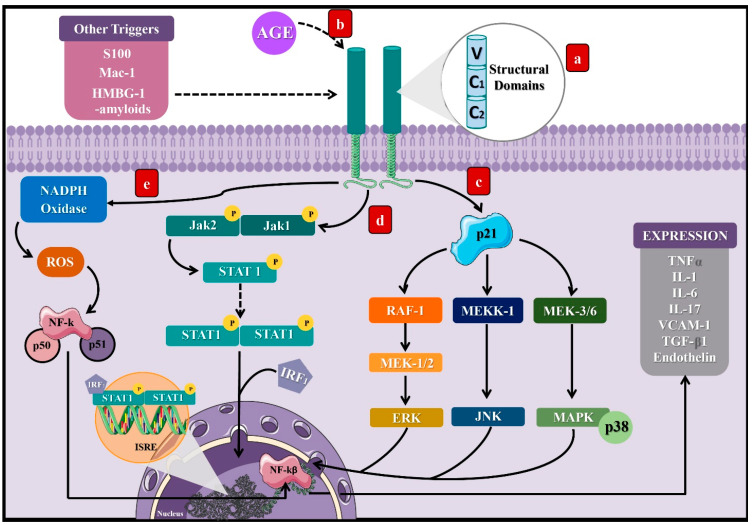
Signaling pathways induced by the activation of the receptor of AGEs. (**a**) RAGE is a cell surface receptor composed of three extracellular domains, a transmembrane domain, and a short cytoplasmic tail. (**b**) AGEs bind to RAGE’s extracellular portion and induce activation of the cytoplasmic domain, leading to different signaling pathways, which will finally result in the stimulation of transcription factors such as NF-KB and ISRE. Three pathways that can lead to such a response: (**c**) activation of the protein p21, which induces RAF-1, MEKK-1, and MEK 3/6 proteins that activate the factors ERK, JNK, and MAPK, which translocate to the cell nucleus. (**d**) Activation of the JAK2/STAT1 pathway, where STAT1 dimerizes and binds to the IRF1 sequence to translocate to the cell nucleus, binding to the ISRE segment and inducing transcription of proinflammatory cytokines. (**e**) Activation of the NADPH oxidase that leads to the stimulation of NF-KB. These processes can be activated by other molecules such as S100, Mac-1, HMBG-1, and β-amyloids. NF-kB: nuclear factor kappa light chain enhancer of activated B cells, ISRE: interferon-sensitive response element, TNFα: tumor necrosis factor-alpha, IL: interleukins, VCAM-1: adherence and growth factors as the vascular cell adhesion molecule-1, TGF-β1: transforming growth factor β, RAF-1: proto-oncogene serine/threonine kinase, MEKK-1: mitogen-activated protein kinase kinase kinase 1, MEK 3/6: mitogen-activated protein kinase kinase, ERK: extracellular signal-regulated kinase, JNK: c-Jun N-terminal kinase, MAPK: mitogen-activated protein kinase, JAK: Janus kinase, STAT: signal transducer and activators of transcription, IRF1: interferon regulatory factor-1, S 100: calgranulin, and HMBG1: high-mobility group box 1.

Additionally, NADH oxidase’s activation directly and indirectly generates reactive oxygen species (ROS) due to RAGE stimulation [37]. Furthermore, RAGE can be activated by other types of ligands aside from AGEs, including S 100 or Calgranulin, Mac-1, High-mobility group box 1 (HMBG1), and β-amyloids, predominantly when their levels increase during inflammatory reactions (Figure 2) [31]. The discovery of RAGE’s multiligand nature explains its elevated and persistent activity in diabetes complications [36,37].

There are different types of these receptors that have been studied, known as soluble forms of RAGE (RAGEs), which are composed of extracellular domains without their intracellular portion, so they can be transported and found free in the plasma [38]. Of note, two types of receptors have been identified, cleaved RAGE(cRAGE) and endogenous secretory RAGE (esRAGE) or RAGE_V1, depending on how they were created [39]. cRAGE is formed from the action of matrix metalloproteases (MMP) and α-disintegrin metalloprotease (ADAM)-10; these enzymes cleave from the cell surface to RAGE, thus losing its transmembrane and cytosolic portions, but it conserves the V1-C1-C2 domains of RAGE. On the other hand, esRAGE is generated by the alternative splicing of the RNAm RAGE gene, changing its structure by adding a 16-amino acid extension at the c-terminal end [40].

Although the distribution and function of these receptors are not yet clear, there are multiple hypotheses about their role in the pathophysiology of inflammatory and metabolic diseases, such as DM. Among the most managed actions, their role as “decoys” for AGEs is evaluated, generating a downregulation effect in inflammation and preventing cell damage due to the sequestration of RAGE ligands that leads to the pathways not activated being intracellularly related to these receptors not only by AGEs but other ligands such as HMB1 or S100, postulating a possible regulatory action on the AGEs–RAGE axis [40]. In addition, studies have established an inversely proportional relationship between the levels of sRAGE and the markers of metabolic syndrome and atherosclerosis in patients with DM, used as a biomarker in inflammatory processes and complications associated with this disease [41].

### 3.1. Molecular Mechanisms of AGEs in Microvascular Complications of DM

The presence of diabetic retinopathy, neuropathy, or (micro) albuminuria defines the existence of microvascular complications of DM [42]; despite affecting different organs, these complications mutually relate to each other [43]. Diverse studies have associated AGEs with the progression of those complications, mainly given the direct action of these products on tissues or via stimulation of the AGE–RAGE axis and the subsequent inflammatory response [44,45,46].

Diabetic kidney disease (DKD) is characterized by renal hypertrophy, proteinuria, decreased glomerular filtration rate, and renal fibrosis [47], ultimately progressing to chronic kidney disease (CDK) [48]. Induction of the AGE–RAGE pathways deriving from the accumulation of these products in renal tissue leads to inflammatory activity [49]. Thus, triggering the migration of macrophages that agglomerate in the renal glomerulus’s mesangium and establishing an inflammatory microenvironment led by IL-6 synthesis with the consequent expansion of this layer eventually causes the compression of capillaries and reduction of the body surface area of renal filtration [50]. Additionally, increased expression of transforming growth factor β (TGF-β) has been correlated with fibrogenesis activation, collagen synthesis stimulation, and renal tubular cell apoptosis, therefore explaining both glomerular sclerosis and dysfunction [51].

**Figure 3:**
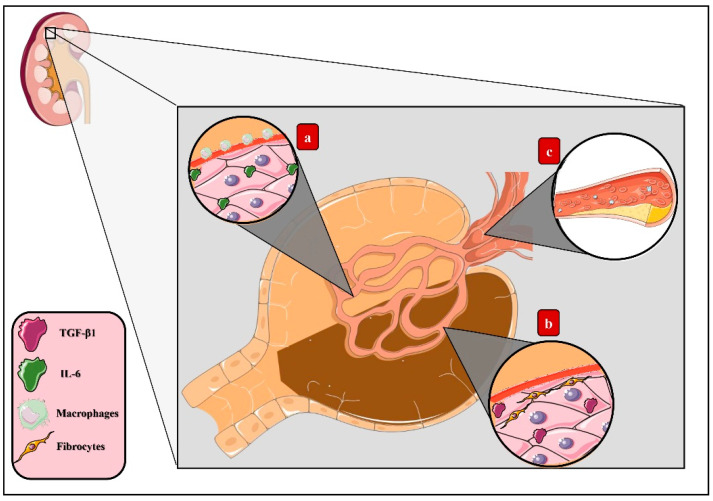
AGEs in diabetic kidney disease. Activation of the AGE–RAGE axis derived from the accumulation of AGEs in renal tissue induces tissue dysfunction through diverse mechanisms: (**a**) macrophage migration, which agglomerates in the renal glomerulus’s mesangium, establishing an inflammatory microenvironment led by IL-6 synthesis and, eventually, causing the expansion of this layer, compression of the capillary, and reduction of the body surface area of filtration. (**b**) Increased expression of transforming growth factor β (TGF-β), which stimulates fibrogenesis, collagen synthesis, and renal tubular cell apoptosis, leading to glomerular sclerosis. (**c**) Lipids storage from altered cholesterol metabolism as a result of the activation of sterol-regulatory element-binding protein 2 (SREBP-2), increasing the expression of 3-hydroxy-3-methylglutaryl-coenzyme A reductase (HMG-CoA reductase) and, finally, concluding in increased cholesterol synthesis.

Other mechanisms induced by the AGE–RAGE pathway arise specifically through CML, which constitutes a higher AGE accumulation in vivo [52], the renal epithelium being continuously exposed to these changes [53]. It has been recently determined that AGEs generate lipid accumulation in this tissue deriving from altered cholesterol metabolism, since AGEs activate the sterol-regulatory element-binding protein 2 (SREBP-2) and, correspondingly, the expression of 3-hydroxy-3-methylglutaryl-coenzyme A reductase (HMG-CoA reductase) increases, concluding in an increased cholesterol synthesis. Additionally, this molecule’s access to cells benefits from the stimulation of low-density lipoprotein (LDLc) activity in conjunction with the decrease of the ATP-binding cassette transporter A1 (ABCA1), which results in tissue dysfunction [44] (Figure 3).

Diabetic neuropathy is defined by the progressive loss of axons within peripheral nerves, clinically manifested by severe pain and sensory impairment [54]. The accumulation of AGEs in the endoneurium, Schwann cells, extracellular matrix, and capillary within these nervous structures cause the glycation of proteins such as fibronectin and laminin [55], inducing structural and functional modifications that decrease the regenerative capacity related to axonal atrophy [56]. Likewise, oxidative stress and, thereby, neuronal cytotoxicity are induced through the AGE–RAGE pathway [57], given the increased levels of superoxide and hydrogen peroxide [58] and decreased intracellular glutathione (GSH) [46], which is an essential antioxidant tripeptide composed of glutamate, cysteine, and glycine [59].

The loss of peripheral sensation and the increase of mechanical pressure in the feet are the primary cause of diabetic foot [60]. Secondly, the oxidative stress, proinflammatory cytokines presence, and glycation of proteins such as collagen lead to the hardening of epithelial cells’ basement membranes, concluding in skin tissue frailty and impaired wound healing [61].

On the other hand, diabetic retinopathy (DR) constitutes a degenerative vascular process that progresses through different stages [62]. First, a blood flow imbalance emerges, in addition to an increased vascular permeability and capillary basement membrane hardening, advancing to the formation of microaneurysms and establishing a microvascular injury that produces ischemia due to decreased retinal blood flow, thus representing a significant cause of blindness [63,64]. The development of these pathological changes results from pericyte apoptosis induced by the AGE–RAGE pathway. Likewise, increased oxidative stress produced by NF-kB expression produces free radicals such as peroxynitrite inside the subretinal membrane and microvasculature, damaging the DNA [65].

Moreover, the regulatory function of Müller cells inside the retina [66] becomes affected in DM by exposure to hyperglycemia [67], and the inflammatory process, alongside its effects on the microvasculature, is also a consequence of activation of the AGE–RAGE pathway [45]. Furthermore, this pathway increases the expression of cytokines and proangiogenic factors such as the vascular endothelial growth factor (VEGF) [68], basic fibroblast growth factor (bFGF) [69], and TGF-β [70], leading to the distinguished neovascularization of DR and significantly exacerbated by the high accumulation capacity of AGEs in the vitreous humor [69].

### 3.2. AGEs and the Macrovascular Alterations in DM

**Figure 4:**
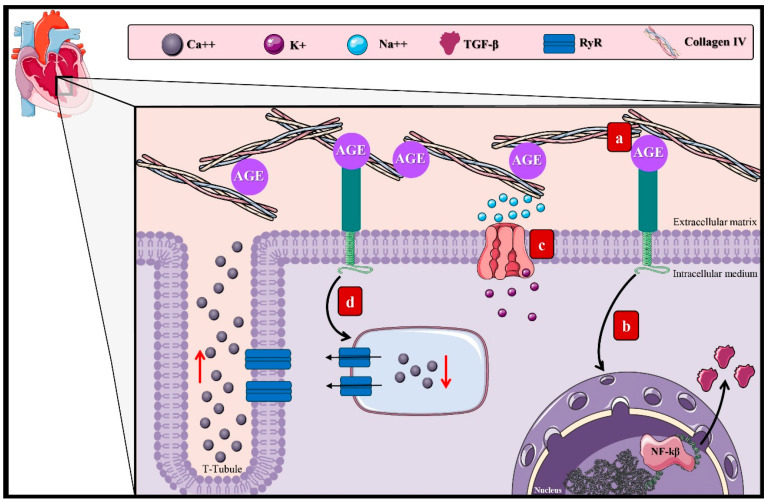
AGEs in diabetic cardiomyopathy. The effect of the AGEs in diabetic cardiomyopathy arises through diverse mechanisms: (**a**) accumulation in the extracellular matrix of cardiac tissue due to the interaction with structural proteins, inducing reticulation between collagen fibers and laminin and decreasing the elastic properties of the cardiac tissue. (**b**) Activation of the AGE–RAGE pathway by the induction of TGF-β and other proinflammatory cytokines that allow the proliferation of fibroblasts conducive to myocardial hypertrophy. (**c**) Activation of the AGE–RAGE pathway and ionic imbalance due to inhibition of the SIRT1/NAD+ pathway, disturbing the function of the Na^++^/K^+^ ATPase. (**d**) Overstimulation of the RyR, generating irregular modifications of the Ca++ levels, favoring its exit and causing an alteration of the cardiac cycle that advances to diastolic dysfunction. Ca^++^: calcium, K^+^: potassium, Na^++^: sodium, TGF-β1: transforming growth factor β, and RyR: ryanodine receptors.

Cardiovascular complications of DM arise as a consequence of the damage to large-diameter vascular structures. They are mostly the leading cause of death among diabetic patients, representing 50% of the deaths related to this disease [27]. Diabetic cardiomyopathy is characterized by ventricular dysfunction originating from myocyte hypertrophy [71,72] and myocardial fibrosis [73]. The AGE–RAGE axis has been admitted as one of the contributing factors to this incompletely elucidated chronic complication (Figure 4) [29].

This process may cause the deterioration of cardiac functions by myocyte hypertrophy [74]. Lately, it has been established that this cardiac remodeling process occurs through the connection within the AGE–RAGE pathway and dendritic cells (DC) [75], which are antigen-presenting cells with essential functions in T-cell regulation and homeostasis [76]. However, it has been reported that the accumulation of matured DC during myocardial infarction could aggravate the tissue remodel [77]. Equally, during in vitro studies, it was determined that the AGE–RAGE pathway promotes DC’s maturation and, therefore, the expression of genes that develop hypertrophy, such as *MYH7*, which encodes the cardiac beta-myosin heavy chain (β-MHC) [75,78].

The increment in fibroblast numbers after the increase of AGEs in the extracellular matrix [74] promotes interactions with structural proteins, inducing reticulation between collagen fibers and laminin, deriving in a loss of the cardiac tissue’s elastic properties, rigidity, and increased cardiac volume, conductive to diastolic dysfunction [79,80]. The AGE–RAGE pathway intervenes in fibroblast proliferation by stimulating proinflammatory genes and TGF-β, amplifying the adverse effect on the cardiac elastic properties [81].

Separately, the accumulation of AGEs in cardiac tissue is also related to the inhibition of the sirtuin-1 protein (SIRT1) expression. SIRT1, a member of the class III deacetylase family, is an antioxidant protein capable of delaying fibrosis and apoptosis of cardiac cells through its activation by NAD^+^ [82]. Besides, the adenosine monophosphate-activated protein kinase (AMPK) keeps a cellular energetic balance and enhances the NAD^+^ levels and can also regulate SIRT1 functions [83]. In conclusion, it has been established that Na^++^/K^+^ ATPase alterations are due to dysregulation of the SIRT1/AMPK pathway, modifying cellular ionic homeostasis [84].

Moreover, the Ca^2+^ levels decrease due to the increased activity of the ryanodine receptors induced by AGE–RAGE [85]. These receptors manage to equilibrate the ion levels during diastole and systole [86]; however, their hyperactivity allows a Ca^2+^ leak from the sarcoplasmic reticulum during diastole, diminishing the Ca^2+^ levels during systole and, thus, disturbing the cardiac cycle [87], driving to cardiac dysfunction [85].

## 4. Progression of Measurement Techniques of AGEs in Patients with DM

Numerous studies have determined the essential role of the plasma levels of AGEs in developing chronic DM complications [29,88,89,90], being described as even better markers than the HbA_1c_ measurements [91]. Experimentally, it has been observed that preventing these products’ accumulation reduces the development and progression of DM-associated complications. As a result, different measurement techniques have been developed to quantify AGEs, including biochemical and immunohistochemical methods capable of measuring products such as pentosidine and CML [92].

The plasma levels of AGEs come from the balance between the synthesis of circulating proteins, accumulation in different tissues [93], absorption from food [14], and renal clearance [94]. For this reason, urine and blood samples allow quantifying their levels that are relevant in DN, especially during the end stage of the disease [95]. The most used measurement technique is the enzyme-linked immunosorbent assay (ELISA), which relies on AGE-recognizing antibodies (Ac), mediated through their bindings and their posterior identification using fluorescence techniques [96]. In this regard, Münch et al. initially described ELISA and validated two procedures: the use of direct monoclonal Ac, which can specifically recognize imidazoline, a product derived from the union of arginine with 3-deoxyglucosone. Secondly, since that technique’s development, different measurement methods using prepared Ac that recognize the epitopes of various AGEs have been designed, including CML [8,96,97].

Nevertheless, applying these types of measurements has been disputed due to both difficulties in reproducing and determining AGE epitopes that can interact with the specific Ac [98]. Thus, the interest in quantifying diabetic patients’ AGEs through their fluorescent properties has increased [21]. Recently, high-performance liquid chromatography (HPLC) analyzes the components of a mixture made by the interaction between the used substances and chromatography columns [99] and can determine the levels of AGEs according to the intensity of their fluorescence. HPLC also allows a faster analysis of protein-bound AGEs such as pentosidine [21] while establishing their influence on the tissues’ biologic characteristics [21].

The use of mass spectrometry (MS) has also been incorporated; this tool allows to identify, characterize, and quantify chemical compounds depending on fragmentation patterns [100]. In this regard, gas chromatography-mass spectrometry (GC-MS) [101] is used as a specific method to quantify oxidative stress markers [102], detecting the activity of products such as CML in heart failure and increasing its association with mortality [101]. Thus, demonstrating the impact of oxidative stress upon these diseases may improve the antioxidant therapy efficacy [103,104]. Likewise, liquid chromatography-MS (LC-MS) [105] has also been developed, granting a more straightforward way to use MS while reducing the limitations presented by GC-MS [100]. This technique allows the determination of plasma levels of AGEs according to DM evolution, exhibiting high levels since the early stages [106], besides their protein-damaging activity in conjunction with other oxidative processes [107], estimating their measure to prevent the development of chronic complications of DM [106].

Despite these techniques’ exceptional results, there are still concerns regarding their cost, complexity, and fluctuations among their results [108]. There is the requirement of newer techniques with more uncomplicated applications and higher reliability [108].

## 5. Measurement of Skin Fluorescence

It has been demonstrated that AGEs heavily conglomerate within tissue structures, specifically inside skin collagen [91]. In this context, a notable difference between tissue and plasma measurement results has been observed [109], the skin collagen being the highest concentration location, thus correlating with the presence and severity of DM’s chronic complications [110]. Hence, the measurement of tissue’s AGEs has become more meaningful, including assessing the cornea, lens [111], or even a skin biopsy [112]. Moreover, there have been techniques designed concerning skin fluorescence and the presence of AGEs in dermis structures [108]. 

In this regard, Meerwaldt et al. developed an instrument capable of measuring skin fluorescence (SAF) in a noninvasive modality and comparing the autofluorescence reader (AFR) results with the results obtained through skin biopsies; they determined its effectivity, validating the application of SAF as a method to quantify AGEs in DM [108].

**Figure 5:**
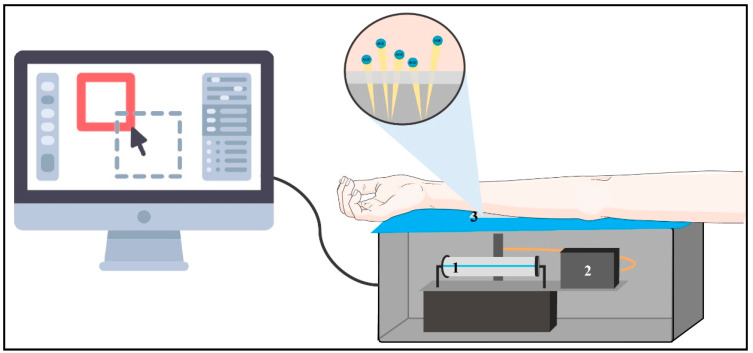
From the surface: the interior of the Skin Autofluorescence Reader. The Skin Autofluorescence Reader consists of a tube that illuminates UV radiation with an intensity of 300–420 nm upon approximately 1 cm^2^ of skin, which must not have scars or other abnormalities. The test must be performed in a semi-dark environment to avoid light interference. Said stimulus excites AGEs, which are bound to skin proteins and have autofluorescent properties within that range. These emit their fluorescence from light waves within a range of 420–600 nm and can be detected by a spectrometer. The autofluorescence of the stimulated AGEs is measured by the proportion between the intensity of the emitted light (420–600 nm) and the excitation light (300–420 nm) multiplied by 100, aiming to compensate for the effect of the skin pigmentation in the capacity of light absorption on the autofluorescence. The obtained results are expressed in arbitrary units (AU) and are shown immediately in a computed connected to the measurer, with a reference established from the results considering the patient’s age. (1) Source of UV light, (2) spectrometer, and (3) illumination window.

The SAF principle relies on the relation between skin fluorescence and the presence of AGEs (Figure 5) [108], explaining its application as a marker to assess the chronic complications of DM [113,114,115,116,117]. Considering that skin collagen’s half-life is about 10–15 years [118], the AGEs bound to skin collagen throughout such a time are a medium to represent the maintained glycemia during long periods [113]. Furthermore, a retrospective study determined that a positive correlation exists between the SAF and HbA1c values measured every three months in patients with DM1, demonstrating a correlation between SAF and long-term glycemic control [119].

While the implementation of SAF to measure the concentration of AGEs in tissue increased, some limitations arose. Among them, skin pigmentation [120], since the measurements in dark skin patients resulted in lower values than patients of Caucasian origin, attributed to the decreased absorption of excitatory or emitted lights from the skin components [121]. For instance, melanin can decrease UV radiation penetration through the epidermis [122], impairing the recognition of fluorescence emitted by AGEs in skin proteins [108].

Based on these findings, Kooetsier et al. validated an algorithm that allows the evaluation of SAF in individuals of different skin colors; contrastingly, this adjustment has solely been applied in healthy individuals [121,123]; in consequence, more research regarding its application on sick patients, mainly with DM, and its complications is required.

## 6. Therapeutic Strategies: Halting the AGE–RAGE Axis

The discovery of the pleiotropic effects of AGEs in the establishment and progression of micro- and macrovascular complications of DM [29] has driven the search for prompt intervention strategies on the advanced glycation pathway that can diminish the damage upon target organs and improve the quality of life of affected patients [124]. Thus, lifestyle modifications and better glycemic control are still the key to prevent the first steps of the AGE process [125]. Indeed, it has been proven an the AGE-rich diet consumption restriction [23,24] added to smoking cessation [126,127] are measurements capable of significantly decreasing the serum levels of CML and MG, with beneficial effects on the skin fluorescence values, respectively. Similarly, it has been reported that an increase of the energy requirements induced by regular physical activity could improve glycemic control and reduce the availability of reactive precursors for glycation reactions, decreasing the accumulation of AGEs in diabetic patients [128].

Nevertheless, over the last few decades, there has been an exhaustive study of pharmacologic agents capable of interfering with the diverse glycation process stages, demonstrating promising results in numerous models in vitro and in vivo [129]. These drugs’ mechanisms of action rely principally on inhibiting the absorption of exogenous AGEs, preventing their endogenous formation and inducing the rupture of preformed AGEs or antagonizing their union with their receptor (Table 1) [130,131,132,133,134,135,136].

### 6.1. Inhibition of the Absorption of Exogenous AGEs

Studying inhibitory compounds of the absorption of AGEs acquires relevance by considering the limitations surrounding the implementation of strict diet modifications to decrease these proinflammatory compounds’ exogenous sources. One particular agent researched to accomplish this issue is AST-120 (Kremezin) [137], an oral adsorbent drug composed of porous spherical carbonic particles mainly indicated for treating patients with CKD to attenuate its progression by removing uremic toxins inside the bowels [138]. AST-120 has been described as capable of binding to food’s CML in the bowels, impairing its absorption. As a result, a significant reduction of the serum levels of AGEs has been reported, aside from decreasing the ARNm of RAGE, the monocyte chemoattractant protein-1 (MCP-1), and the vascular cell adhesion molecule-1 (VCAM-1) in endothelial cells, diminishing the inflammatory response induced by these molecules [139]. On the other hand, sevelamer carbonate, a nonabsorbable oral drug used primarily as a phosphate binding agent to prevent hyperphosphatemia in patients with CKD [140], has proven to prevent the absorption of diet AGEs, in addition to decreasing the serum and cellular levels of AGEs and other inflammation markers [141].

### 6.2. Inhibition of the Endogenous Formation of AGEs

To date, the most widely studied drugs are precisely those destined to impede endogenous AGE formation. The first drug developed to achieve this purpose was aminoguanidine (AG) [142], a compound of hydralazine that, in virtue of its guanidine group, is capable of trapping α-dicarbonyl compounds such as MG and 3DG produced during the glycation early stage and, therefore, can prevent their subsequent reaction with amino groups of proteins [143,144]. The outcome described above has been correlated to a significant attenuation of chronic complications of diabetes [145], such as atherosclerosis, DN, retinopathy, and neuropathy, in numerous experimental studies [146,147]. Accordingly, studies have focused on elucidating the inhibitory properties of the AGE formation of bioactive compounds derived from herbs and spices [148], along with multiple drugs used in clinical practice—specifically, for instance, metformin [149], the vitamin B complex, and even antihypertensive drugs such as the angiotensin-converting enzyme inhibitors (ACEI) and angiotensin II receptor blockers (ARBs) [150].

Concerning phytochemicals, through the inhibition of AGE precursors, experimental studies have determined an antiglycation effect of polyphenols and other bioactive compounds extracted from plant species such as *Cinnamomum verum J.* (Ceylon-Cinnamon), *Syzygium aromaticum L.* (Cloves), *Pimpinella anisum L*. (Anise), *Pimenta dioica L*. (Allspice), *Rumex japonicus*, *Ilex paraguarieusis*, *Piper auritum*, and *Origanum majorana* [151,152,153,154]. Although the mechanisms behind the antiglycation effect are not precisely known [155], it has been reported that compounds such as resveratrol, oxyresveratrol, and piceatannol can inhibit the production of AGEs through the elimination of reactive carbonyl species [156].

Metformin, a biguanide extensively used as an antihyperglycemic drug for treating patients with DM2 [157], has recently been linked to important antioxidant and anti-inflammatory properties [158,159]. Using metformin as an inhibitor of AGEs is justified due to its structural similarity to AG, and it has also been proven capable of reacting with α-dicarbonyl compounds [160], preventing the posterior production of AGEs [161].

Contrarily, vitamins such as pyridoxamine (PM), thiamine pyrophosphate, and its lipophilic derivative benfotiamine have been demonstrated to intervene during the late stage of the glycation process, blocking the transformation of Amadori products into AGEs [162,163]; however, their actions are carried out throughout different levels. PM traps reactive oxygen species, leading to the blockage of the oxidative degradation of Amadori intermediaries while promoting the elimination of toxic carbonyl products originating from glucose and lipid degradation [164,165,166]. Differently, thiamine pyrophosphate and benfotiamine increase the transketolase activity, an enzyme that reduces the accumulation of glycolytic metabolites like glyceraldehyde-3-phosphate and fructose-6-phosphate by stimulating the pentose phosphate pathway, both involved in the formation of intracellular AGEs [163].

Another form to impede the formation of AGEs depends on the chelation of transition metals [167] such as Mg^2+^, Cu^2+^, and Zn^2+^, since, during hyperglycemic conditions, these function as catalysts of oxidation reactions, thus favoring the formation of AGEs [168]. Regarding this, it has been found that the inhibitory actions on the formation of AGEs shown by some antihypertensive agents such as losartan and valsartan [169,170] can be attributed to their antioxidant and metal-chelating properties, which have also been associated with the decreased plasma levels of AGEs in various experimental studies [171].

### 6.3. Breakage and Reversal of Preformed AGEs

Other sorts of compounds have obtained relevance throughout the search for inhibitors of the glycation pathway. These are the *N*-phenacylthiazolium bromide (PTB) [172] with its derivative ALT-711 or alagebrium (dimethyl-3-*N*-phenacylthiazolium chloride) [173], given their ability to cleave AGE-AGE crosslinks that maintain AGEs attached to tissue proteins like collagen and elastin [174]. Thoroughly, their precise mechanisms of action rely on their reactions to carbonyl groups located in the crosslinks between AGEs, subsequently promoting the spontaneous cleavage of carbon–carbon bonds at physiologic pH [175]. Likewise, an experimental study demonstrated the efficacy of MnmC, an enzyme involved in bacterial tRNA modification, capable of performing a catalytic reversion of the AGEs carboxyethyl-lysine (CEL) and carboxymethyl-lysine (CML) to lysine’s native structure [176]. The application of these drugs, mainly alagebrium, in diverse animal models has proven to be helpful in arterial stiffness reduction, blood vessel fibrosis [173,177], the development of atherosclerosis [178], cardiovascular disease [179], hypertension [180], and kidney injury [181].

### 6.4. Antagonism towards RAGE-Binding

Finally, agents capable of antagonizing the binding to RAGE could inhibit the harmful effects of AGEs. This antagonism could function through different means, such as inhibiting RAGE expression, interfering with intracellular signaling mediated by RAGE, or increasing the plasma levels of the circulating sRAGE, given its ability to serve as a decoy receptor to trap AGEs [182,183]. Numerous existing agents have proven to reach these objectives; among the most remarkable are statins [184,185] and thiazolidinediones [186]. Beyond their potential as a lipid-lowering agent [187] and an oral hypoglycemic agent, respectively [188], both have demonstrated an in vivo reduction of RAGE expression [184,185,186] in conjunction with increased serum levels of sRAGE. It has been suggested that said effects depend upon the activation of the peroxisome proliferator-activated receptor γ (PPAR-γ), which could inhibit the phosphorylation of ERK1/2 and, thus, suppress the activation of NF-KB, decreasing the expression of both proinflammatory cytokines and RAGE [189,190]. Moreover, azeliragon (PF-04494700 or TTP488), an oral antagonist of RAGE, has obtained favorable results in animal models of Alzheimer’s disease and was proven safe and effective at low doses in diverse clinical trials [191,192] (NCT02080364), being established as a promising therapeutic agent in the management of chronic complications of DM such as diabetic retinopathy [193].

Other molecules being widely researched are glucagon-like peptide-1 (GLP-1) and its analog exendin-4. Various experimental studies have shown their ability to decrease RAGE expression by suppressing NF-KB and reducing ROS generation by decreasing NADPH oxidase activity [194,195]. Consequently, these results have been associated with reducing the damage related to the activation of the AGE–RAGE axis in diseases like diabetic retinopathy [196], atherosclerosis [197], and diabetic cardiomyopathy [198].

## 7. Future Perspectives

The clinical potential of these interventions has not been completely established yet. For instance, aminoguanidine, despite being the first drug capable of examining the concept that inhibiting the formation of AGEs could lead to a clinically significant attenuation of a severe complication of diabetes [199], had to be suspended due to drug safety issues secondary to multiple adverse effects, such as its prooxidative potential [200], inhibition of NO synthase [201], abnormalities in liver function, development of antineutrophil cytoplasm antibodies, and even some cases of glomerulonephritis [145].

However, some agents, including alagebrium, benfotiamine, pyridoxamine, and thiazolidinediones, have achieved promising results in recent clinical trials [130,131,132,133,134,135,136,202,203,204]. Nonetheless, there are no available therapies specifically directed to prevent or eradicate AGEs; most of the preliminary clinical trials have focused on assessing the single effect of any particular AGE inhibitor, excluding the rest of the signaling pathways that could be intervened. Respectively, it would be excellent to research the combined effect of two or more inhibitors that act on different steps of the AGE–RAGE axis. Additionally, designing a drug with a broad spectrum of action to obtain better results in controlling the adverse effects of AGEs in the organism can be a further alternative.

In this sense, sRAGE has emerged as a promising molecule to competitively inhibit RAGE activation, modulating the systemic inflammation produced by the AGE–RAGE axis in multiple experimental studies. [205]. By acting as a decoy for RAGE ligands, sRAGE is able to attenuate inflammatory signaling, oxidative stress, and metabolic dysregulation. However, these findings came from experimental models [206].

## 8. Conclusions

The action of AGEs in the development of chronic complications of DM has been reported. The increase in plasma concentrations of AGEs, either due to an increase in their consumption or due to their intrinsic formation, as well as the subsequent activation of RAGE, is known as the AGE–RAGE axis, which functions as a powerful inducer of proinflammatory pathways by promoting the synthesis of cytokines such as TNF-α, IL, VCAM-1, and even the increase of ROS, which has harmful effects on the micro- and macrovasculature of the target organs affected in DM. As a result, the interest in AGEs as a therapeutic target has increased, with the aim of creating drugs that can interfere with the activity of these molecules, decrease their concentration, or antagonize the AGE–RAGE axis, opening up the possibility of finding a new way to reduce the damage in target organs and improve the quality of life of patients. Additionally, the measurement of AGEs has acquired relevance, since they can be considered better markers than HbA1c. Studies using the SAF assessment have shown that increased levels are related to the risk factors of long-term complications; thus, these noninvasive tools could be used to establish the risk of developing DM complications, allowing for prompt intervention.

## Figures and Tables

**Table 1:** Recent clinical evidence of therapeutic interventions in advanced glycation products and their complications.

Mechanism of Action	Drug	Methodology	Results	Author [REF]
Inhibition of the endogenous formation of AGEs	Benfotiamine and Pyridoxamine	Randomized, double-blinded controlled by placebo trial, which included 30 patients with primary osteoarthritis divided randomly between two groups to receive tablets of inhibitors of AGEs (benfotiamine (50 mg) + PM (50 mg) + methylcobalamin (500 mg)) or placebo tablets, three times a day.	Significant decrease in serum levels and fluorescence of AGEs. Decreased pain and inflammation. Increase in daily activity and mobility in patients with osteoarthritis.	Garg S et al. (2013) [130]
	Pioglitazone and Metformin	Randomized, open parallel-groups trial, performed in patients recently diagnosticated with DM2, who were given 30 mg/day of pioglitazone (n = 30), 1000 mg/day of metformin (n = 50) or not any drugs (n = 49).	In both treated groups with either pioglitazone or metformin was observed a statistically significant decrease in levels of AOPP and AGEs, besides causing an increase in FRAP (a marker of plasma antioxidant capacity).	Mirmiranpour H et al. (2013) [131]
	Enalapril and Lercanidipine	Randomized, double-blinded trial, which included 359 ambulatory patients <65 years of age, first-diagnosed with essential hypertension and without treatment, divided between three groups, who were randomly given: enalapril 20 mg/day (n = 126), lercanidipine 10 mg/day (n = 115), or enalapril + lercanidipine 20/10 mg/day (118), in order to assess their effects in markers of cardiovascular risk.	All treatments showed a significant increase in levels of sRAGE, which was higher in patients treated with enalapril + lercanidipine.Significant reduction of levels of TNF-α and US-CRP in patients treated with enalapril + lercanidipine	Derosa G et al. (2014) [132]
	Benfotiamine	Randomized, double-blinded, controlled trial, which included 41 patients with DM2 without complications, who were randomly given 900 mg/day of benfotiamine or 900 mg/day of a placebo, to assess their effect on levels of AGEs and sRAGE.	Patients treated with benfotiamine had a statistically significant decrease in levels of carboxymethyl-lysine. There were no statistically significant differences in levels of sRAGE.	Contreras C et al. (2017) [133]
Breakage and Reversal of preformed AGEs	Alagebrium (ALT-711)	Randomized, double-blinded, controlled by placebo prospective study, performed in 57 healthy subjects over 60 years of age, which were randomly divided between 4 groups: sedentary + placebo, sedentary + alagebrium (200 mg/day), exercise + placebo, and exercise + alagebrium in order to assess their effect in hemodynamics, function, and structure of the left ventricle.	Alagebrium led to a moderate improvement in rigidity of the left ventricle, which was more prominent when it was combined with physical activity.	Fujimoto N et al. (2013) [134]
	Alagebrium	Randomized, controlled by placebo trial, which included 47 older subjects previously sedentary, who were divided between 4 types of interventions: Exercise + Alagebrium (200 mg/day), Exercise + Placebo, Alagebrium (200 mg/day), and Exercise aiming to examine their effect over endothelial function, arterial stiffness, and cardiovascular risk.	There were no improvements in endothelial function or arterial stiffness in any of the four groups.	Oudegeest-Sander M et al. (2013) [135]
Inhibition of the absorption of exogenous AGEs	Sevelamer carbonate	Randomized, open, single-blinded trial, which included 117 patients with T2DM and diabetic nephropathy in stages 2 to 4, who were given sevelamer carbonate (1600 mg) or calcium carbonate (1200 mg), three times a day, to measure their effects in the AGE–RAGE axis and onto oxidative stress.	Patients treated with sevelamer carbonate showed a significant reduction of both circulating and intracellular AGEs (carboxymethyl-lysine and methylglyoxal).It was also observed a significant increase in antioxidant defenses and reduction of pro-oxidant molecules.	Yubero-Serrano et al. (2015) [136]

AGEs, advanced glycation end products; AOPP, advanced oxidation protein products; T2DM, Type 2 diabetes mellitus; FRAP, Ferritin reducing ability of plasma; HbA1C, glycated hemoglobin; RAGE, receptor for advanced glycation end products; sRAGE, soluble RAGE; TNF-α, tumor necrosis factor-α; US-CRP, ultra-sensitive C-Reactive Protein; and sVCAM, soluble vascular cell adhesion molecule-1.
